# Neuromuscular factors predicting lower limb explosive strength in male college sprinters

**DOI:** 10.3389/fphys.2024.1498811

**Published:** 2025-01-07

**Authors:** YanJin Li, QiaoFeng Guo, Jia Shao, YanMing Gan, YaJing Zhao, Yue Zhou

**Affiliations:** ^1^ Sports Science School, Beijing Sport University, Beijing, China; ^2^ Beijing Research Institute of Sports Science, Beijing Municipal Bureau of Sports, Beijing, China; ^3^ China Athletics College, Beijing Sport University, Beijing, China; ^4^ The “Belt and Road” Joint Laboratory of Winter Sports, Beijing Sport University, Beijing, China

**Keywords:** H-reflex, V-wave, sprinter, neuromuscular, stiffness

## Abstract

**Purpose:**

This study aimed to explore the effects of neural and muscular factors on lower limb explosive strength in male college sprinters, and build models based on those factors to identify the key neuromuscular factors that predict the rate of force development (RFD) and 30 m sprint time.

**Method:**

15 male college sprinters were recruited in this study, with 100 m personal best times under 10.93 s. The neuromuscular data were collected by H-reflex and V-wave, isokinetic muscle strength, vertical jumps, and 30 m sprint tests. Pearson correlation and multiple stepwise regression were used for data analysis. The level of statistical significance was set at *p* ≤ 0.05 for all analyses.

**Results:**

30 m sprint time had a significant moderate positive correlation with Achilles tendon stiffness (*r* = 0.50, *p* = 0.05, 95%CI: 0.01–0.81) and a significant moderate negative correlation with the H-index (*r* = −0.54, *p* = 0.04, 95%CI: 0.82 to −0.03), V wave (V/MmaxA, *r* = −0.59, *p* = 0.02, 95%CI: 0.85 to −0.11) and the eccentric strength of Hamstring (HECC, *r* = −0.53, *p* = 0.04, 95%CI: 0.82 to −0.03). The rate of force development (RFD) had a significant positive correlation with H reflex (Hmax/Mmax, *r* = 0.57, *p* = 0.03.95%CI:0.08–0.84), and the eccentric strength of Quadriceps (QECC, *r* = 0.53, *p* = 0.04, 95%CI: 0.02–0.82). V/MmaxA and HECC were identified as predictors of 30 m sprint time, and the *R* ^2^ explained 57.5% of the variance. Vertical stiffness and QECC explained 82.7% of the variation in the RFD.

**Conclusion:**

This study found that V/MmaxA and HECC were predictive factors of 30 m sprint time, vertical stiffness and QECC were the predictive factors of RFD. Neural factors such as the α-motoneurons excitability of the spinal and supraspinal centers, have a greater influence on lower limb explosive strength in male college sprinters. Therefore, training related to the neural function of sprinters should be emphasized. In addition, H reflex and V wave can be used widely to assess and monitor the neural function of sprinters in future research. The impact of neural drive on muscles in different levels and sexes of sprinters, and the neuromuscular modulation during muscle contractions can be further explored.

## 1 Introduction

Sprinting is one of the fastest events in the Olympic Games, the world record for the 100 m sprint is 9.58 s created in 2009 (Haugen et al., 2019; Kawama et al., 2024). The sprinters need to start rapidly and accelerate to maximal velocity (Donaldson et al., 2022). Acceleration is one of the main phases in the 100 m or 200 m race, which is critical to increasing maximal horizontal velocity and sprint performance (Healy et al., 2022; Nagahara et al., 2014). A 30-m (30 m) sprint test is a classical method of assessing acceleration, which requires sprinters to accelerate rapidly from zero to a high horizontal velocity, with a strong correlation with 100 m performances (Bezodis et al., 2015; Bezodis et al., 2019; van den Tillaar et al., 2023). The best sprinters have a higher level of acceleration and require a high level of lower limb explosive strength (Slawinski et al., 2017).

Lower limb explosive strength refers to sprinters’ ability to generate maximum force in a very short time, helping them achieve good acceleration (Samozino et al., 2022; Watanabe, 2018). Studies have demonstrated that elite sprinters have greater lower limb explosive strength, with a higher rate of force development (RFD) than sub-elite sprinters (Crotty et al., 2024; Slawinski et al., 2010). Lower limb explosive strength is regulated by the central nervous system, where neural impulses are transmitted through nerve fibers to the anterior horn α-motor neurons of the spinal cord. This process excites α-motor neurons, rapidly recruiting their associated muscle fibers for explosive contraction. Therefore, it is speculated that lower limb explosive strength is affected by neural factors and muscular factors, such as rapid recruitment speed of α-motor neurons, high motor unit discharge rates, and fast muscle fiber contractions (Dideriksen et al., 2020; Del, 2023). A study found that RFD increased after 4 weeks of strength training can be attributed to the enhanced recruitment speed of α-motor neurons (Del et al., 2022).

Previous studies have predominantly used reaction time to assess the neural function of sprinters (Tonnessen et al., 2013). However, the neural function of sprinters can be explored using more detailed methods. H reflex and M wave were the effective and non-invasive methods to reflect the spinal neural function, induced by electrically stimulating the peripheral nerve (mixed nerve), founded by German physiologist Paul Hoffmann in 1918 (Magladery and Mcdougal, 1950). The reflex arc of the H-reflex is similar to the muscle stretch reflex. H reflex was evoked by the electrical stimulation of Ia afferent nerves (bypassing the muscle spindle), which activates spinal motor neurons and recruits motor units, resulting in muscle contraction (Wolpaw, 2010). M wave was the compound muscle action potential, indicating the direct response of the muscle to electrical stimulation of the motor nerve (bypassing the reflex pathway) in H reflex tests (McNeil et al., 2013). The action potentials of the H reflex and M wave can be recorded via surface electromyography (EMG). Therefore, H-reflex and M wave were used to assess the excitability of the spinal α-motor neurons and the synaptic transmission efficiency of the Ia afferent, which also reflected the plasticity of the spinal cord (Aagaard et al., 2002; Theodosiadou et al., 2023). In recent studies, the H reflex was widely to assess spinal cord function across various populations, such as patients with spinal cord injury, healthy people, and athletes (Guan and Koceja, 2011; Raffalt et al., 2015; Sun et al., 2022; Vila-Chã et al., 2012).

Animal experiments identified that the plasticity of the spinal cord is primarily trained through the corticospinal tract (de Leon et al., 1998). The spinal cord is the final common pathway of the motor pathway, receiving sensory and motor signals from the central and peripheral nervous systems. V wave is an electrophysiological variant of the H-reflex, which reflects the excitability of descending corticospinal pathways, and the supraspinal input to the spinal motoneuron pool (Aagaard et al., 2002). H reflex and M wave were evoked by submaximal electrical stimulation of the peripheral nerves in rest, and V wave was evoked by supramaximal electrical stimulation of the peripheral nerves during maximal voluntary contractions (MVC) (McNeil et al., 2013). V wave was the action potentials recorded after orthodromic impulses from descending voluntary drive collided with antidromic impulses evoked by supramaximal stimulation of motor axons. Studies have shown that strength-trained athletes have lower H-reflex excitability thresholds and higher V-wave responses than untrained individuals (Grosprêtre et al., 2018; Tøien et al., 2023; Vila-Chã et al., 2012). Changes in the H-reflex and V-wave were observed after resistance training, accompanied by enhanced sports performance (Aagaard, 2018; Holtermann et al., 2007; Kinnunen et al., 2019a).

Muscle-tendon function is fundamental to the development of lower limb explosive strength in sprinters (Cavedon et al., 2023). One hundred-meter (100 m) sprinters with more lean body mass and strength significantly have better sprint performance (Barbieri et al., 2017). The biomechanics data from a full-body musculoskeletal model for predicting sprint performance showed that sprint was most sensitive to changes in muscle (Lin and Pandy, 2022). An investigation revealed that the muscle group sprint coaches considered to be the most important for enhancing lower limb explosive strength are quadriceps, hamstrings and gastrocnemius (Healy et al., 2021). Sprint mechanical parameters changed after 5 months of training in national-level sprinters, accompanied by increments of muscle volumes in the lower limb (Nuell et al., 2020). Tendon also plays an important role in sprint, the ankle and plantar flexor muscle-tendon units rapidly apply force to the ground by storing and releasing elastic energy, shortening the contact time to maintain high velocity (Crotty et al., 2024; Yamazaki et al., 2022). Sprinters are characterized by higher active tendon stiffness during high-speed movements after training (Kubo et al., 2020).

Most studies have researched the effects of neural and muscular factors on lower limb explosive strength in sprinters, but determining which factor plays a more critical role still requires further investigation. In addition, studies investigating neural function in sprinters through H-reflex and V-wave analysis are relatively limited. Thus, this study aimed to explore the key neuromuscular factors predicting lower limb explosive strength, focus on assessing the neural function from spinal and supraspinal in sprinters through the H reflex and V-wave, and hypothesized that neural factors have a greater influence on lower-limb explosive strength.

## 2 Methods

### 2.1 Subjects

Fifteen male college sprinters (age, 19.73 ± 1.39; height, 181.40 ± 4.32 cm; weight, 70.98 ± 4.21 kg; lean body mass, 36.05 ± 1.13 kg) volunteered for the study and signed written informed consent. This study has been approved by the university’s ethics committee (No. 2024096H).

All subjects had more than 5 years of training experience. Inclusion criteria required: (1) the subjects should be national level-1 athletes (100 m personal best times under 10.93 s in national or provincial competitions); (2) maintain routine technical and strength training sessions, no training interruption; (3) no neurological disorders or musculoskeletal injuries in the past 6 months before testing; (4) no medication to enhance sports performance was taken during the tests.

All subjects have a familiarization session before tests. The subjects conducted H reflex and V wave tests, followed by muscle and tendon function tests, 30 m and CMJ tests, with the interval between each test at least 48 h.

### 2.2 H reflex and V wave

H-reflex and V-wave were elicited by a constant current electrical stimulator (DS7A, Digitimer, England). The subjects were positioned prone on the bed, previous studies found that the prone position is the best position to evoke the H-reflex (Cecen et al., 2018; Jeon et al., 2007). During the phase of electrical stimulation, the subjects need to keep their heads fixed to avoid vestibular effects on motor neurons and keep their body relaxed (Kennedy and Inglis, 2002). A handheld electrode was placed in the popliteal fossa of the right lower limb to test the optimal stimulation site, and then a disposable self-adhesive electrode was applied as the cathode. Previous studies found no interlimb differences in short-distance athletes (Ozmerdivenli et al., 2002; Sun et al., 2022). A 5 × 8 cm disposable self-adhesive electrode was placed over the patella as the anode.

Action potentials evoked by electrical stimulation were recorded by surface electromyography (EMG) electrodes (Trigno Wireless EMG System, Delsys, United States) sampled at 2000 Hz. The electrodes were placed in the middle of the muscle belly of the right lateral gastrocnemius (LG) muscle and the tibialis anterior (TA), with the hair removed and the skin surface cleaned. The longitudinal plane of the electrodes was aligned with the muscle fibers (Tagore et al., 2023). In our preliminary experiments, we found that sprinters more easily evoke the H-reflex in the LG muscle in a prone position.

**FIGURE 1:**
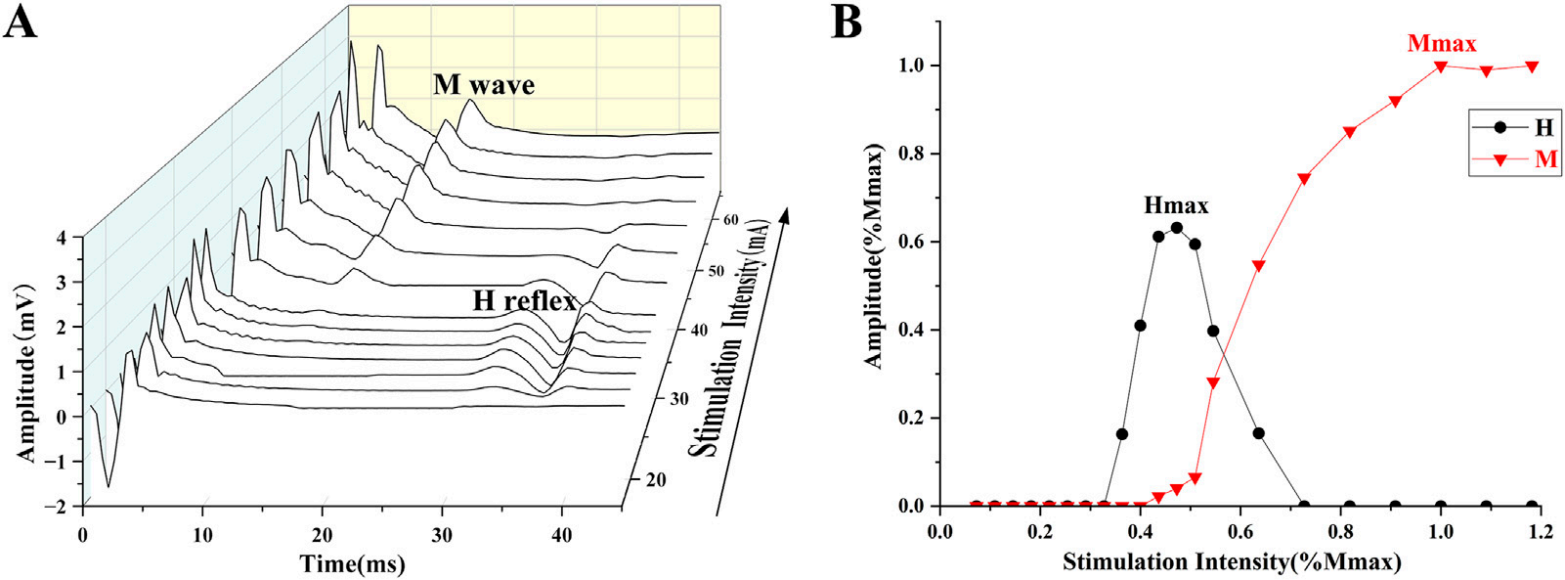
**(A)** The process of evoking the H-reflex and M-wave. **(B)** H-reflex and M-wave normalized recruitment curves.

**FIGURE 2:**
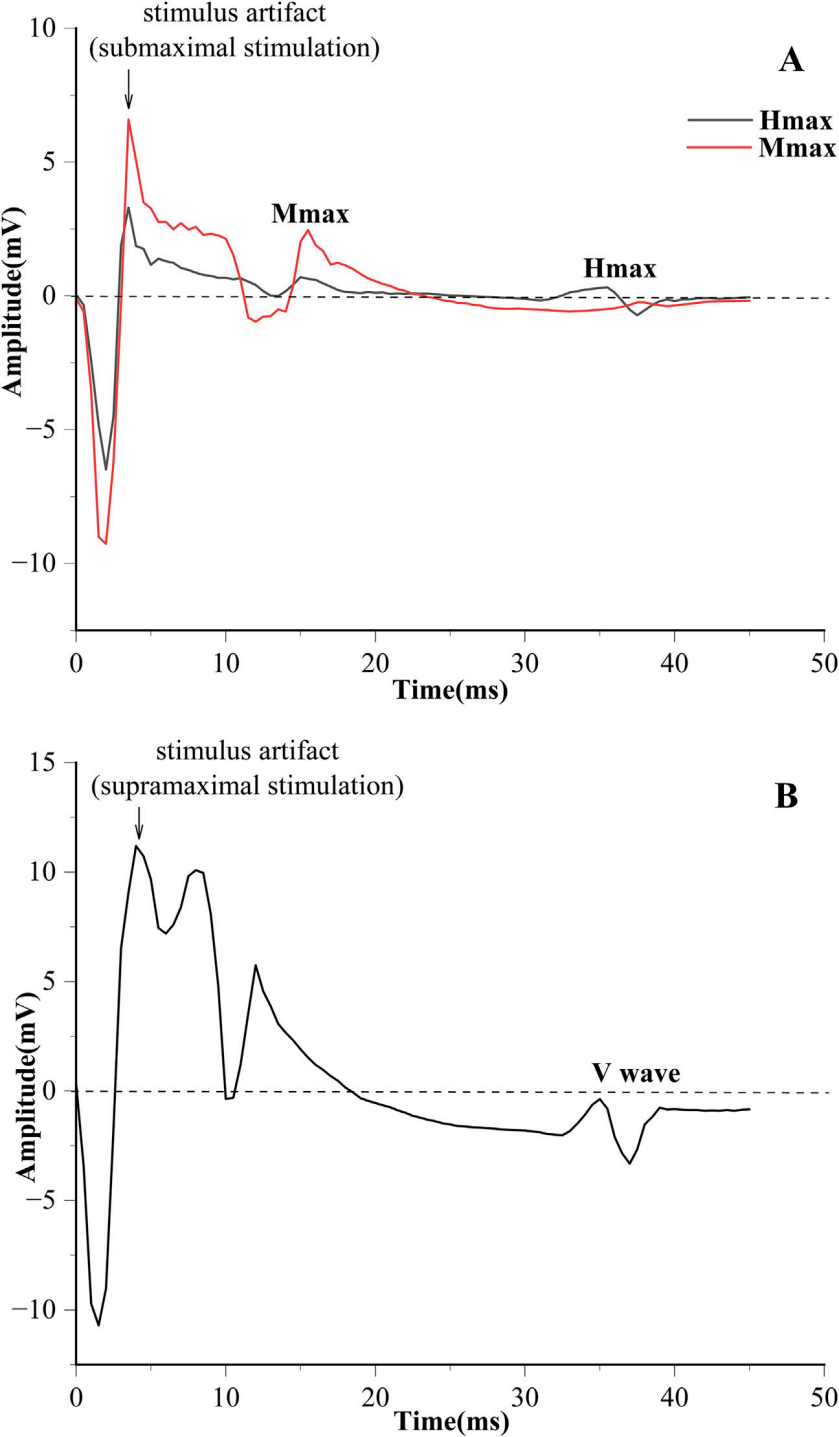
Responses to electrical stimulation evoking Hmax and Mmax **(A)**, and Vmax **(B)**.

Electrical stimulation was used to obtain the H-reflex and M-wave recruitment curves (Figure 1). The square pulse was set to 1,000 μs (Theodosiadou et al., 2023). The stimulation started at 4 mA, with increments of 2 mA per stimulation until the maximum H-reflex (Hmax) was evoked. Subsequently, each stimulation was increased by 5 mA until the maximum M-wave (Mmax) was observed (Vila-Chã and et al., 2012). The interval of each stimulation is 10 s (Kipp et al., 2011). V-wave was elicited by electrical stimulation with 150% Mmax (MmaxA) during maximum voluntary contraction (MVC) contraction (Figure 2) (Aagaard, 2003).

### 2.3 Muscle and tendon function

The lower limb muscle strength of the knee joint was assessed by an isokinetic dynamometer (IsoMed 2000; D. and R. Ferstl GmbH, Hemau, Germany), and high reliability has been demonstrated in previous studies (Zhou et al., 2022; Zoger et al., 2023). The parameters examined included peak torque (PT) of the hamstring (H), quadriceps (Q), gastrocnemius muscle (GM), and the H/Q ratio of the dominant leg during concentric (CON) and eccentric (ECC) contractions at slow (60°/s) and fast (180°/s) speeds. Only a slow speed is conducted under ECC to prevent muscle strains in sprinters (Brigido-Fernandez et al., 2022). A 10-minute warm-up was performed before the test. The subjects sat on a dynamometer, and the shoulder, pelvis and thigh were secured comfortably with padded straps to minimize compensatory movements. The knee joint was aligned with the dynamometer axis of rotation, and gravity compensation was turned on (Brown et al., 2014). Each test includes 2 sets; each set has 5 repetitions, with a 1-min break between each test. The PT results were normalized to body weight (Nm/kg).

Tendon stiffness was used to evaluate tendon function and elastic properties in athletes, correlated with the ability to increase force production during explosive contractions (Bojsen-Moller et al., 2005; Radovanovic et al., 2022; Sukanen et al., 2024). Tendon stiffness was measured in the ankle joint using MVC and ultrasound, calculated as the ratio of PT and the displacement of the Achilles tendon (Arampatzis et al., 2007). The subjects were prone on IsoMed 2000, with the right foot fixed on the pedal, and the PT of their plantar flexor muscle in isometric contraction was tested. The MVC test required the subjects to develop a gradual increase in torque from zero (at rest) to maximum effort (MVC) for 5 s (Kubo et al., 2000). The displacement of the Achilles tendon was visualized and measured by a B-mode ultrasound (MuscleSound LOGIQe, GE, United States). A 60 mm linear array probe (12 MHz scanning frequency) was positioned at the GM muscle-tendon junction to record displacement, which was subsequently analyzed using embedded software. Pressure between the probe and the skin was maintained at a minimum level.

### 2.4 30 m and CMJ tests

Parameters of kinetics and kinematics in the countermovement jump (CMJ) were measured using an eight-camera 3D motion capture system (Arqus A12, Qualisys, Sweden, 200 Hz) and a force plate (9286ba, Kistler, Switzerland, 2000 Hz). To track the motion of the subjects, 36 retro-reflective markers (14 mm in diameter) were affixed to the subjects’ whole body, following the Helen- Hayes model (Thompson et al., 2024; Zamporri and Aguinaldo, 2018).

After warming up, the subjects performed CMJ 2–3 times with submaximal effort, the interval of each jump was 1 min (Douligeris et al., 2023). Subsequently, the subjects performed standard CMJ with maximal effort on the force plate at least 3 times, with their hands on their hips (Fristrup et al., 2024). There was a 3-minute rest between each jump. The vertical ground reaction force (vGRF) was recorded (McLellan et al., 2011). Kinematic data were tested by the Qualisys Track Manager system, and processed by Visual 3D software (C-Motion, United States) to establish a virtual center of mass point, obtaining the vertical displacement of the center of mass (ΔCOM) during CMJ. Vertical stiffness was calculated by the ratio of vGRF (the difference between the peak value and the minimum value) to ΔCOM during the eccentric phase of CMJ, reflecting the storage and release of potential elastic energy via the musculotendinous unit in the stretch-shortening cycle (SCC) (Meyer et al., 2023; Satkunskiene et al., 2021).

Previous studies showed that 30 m and RFD were regarded as reliable indicators reflecting lower limb explosive strength and acceleration performance in sprinters (Loturco et al., 2021; Morin et al., 2022; Pantoja et al., 2016). The subjects performed the 30 m test twice, with a 5 min rest interval, which was tested by a timing system (Smart speed PT, Fusion Sport, Australia). The 30 m sprint time was recorded as the time duration taken by the subjects to pass through two automated light gates. The RFD derived from the force-time curves obtained during the minimum to the maximum phase of eccentric force in the CMJ, which assesses the lower limb explosive strength and neuromuscular function (Maffiuletti et al., 2016; Yamauchi and Koyama, 2020). The RFD was normalized by body weight.

### 2.5 Data and statistical analyses

EMG signals were calculated using Delsys software, with embedded band-pass filters of 20–450 Hz and the magnitudes of H-reflex, M-wave, and V-wave were calculated with the peak-to-peak amplitude of the corresponding signals (Sun et al., 2022). The results of H-reflex and V-wave are generally presented as amplitude values, normalized to Mmax and MmaxA respectively (H/M, V/MmaxA). The recruitment curves of H-reflex, M-wave, and V-wave are shown in Figure 1.

The thresholds of H-reflex and M-wave were recorded and calculated in the Hth/Mth. The latencies of the H-reflex (Hlat) and M-wave (Mlat) were measured from the stimulus artifact to the onset of the potential and used to calculate the H-index, which reflects the nerve conduction velocity (NCV) (Melo et al., 2023). H-index is calculated as the equation: ${\left[\frac{Body\;Height}{Hlat-Mlat}\right]}^{2}*2$ (Kim et al., 2022).

SPSS software (26.0, IBM, United States) was used for the statistical analysis and calculations. Shapiro–Wilk tests were used to examine the normality of the variables. Pearson’s correlation coefficient was used to analyze the relationship between lower limb explosive strength and neuromuscular factors. The *r* value was interpreted as little (r < 0.25), small (0.25 ≤ r < 0.50), moderate (0.50 ≤ r < 0.75), and strong (r ≥ 0.75) (Portney, 2009).

A stepwise multiple linear regression model was built to determine the strongest predictor of the lower limb explosive strength of sprinters. The variables showed a strong correlation with the 30 m was selected as the independent variable and included in the multiple regression model to find the key factors that could predict lower limb explosive strength. VIF < 5 was considered indicative of the absence of multicollinearity. The 95% confidence interval (CI) was reported. The level of statistical significance was set at *p* ≤ 0.05 for all analyses.

## 3 Results

The descriptive values of the H-reflex and V-wave, skeletal muscle function, and lower limb explosive strength are shown in Table 1.

**TABLE 1:** Descriptive statistics of H reflex, V wave, and skeletal muscle function (mean ± SD).

Variable			Value	95%CI
H-reflex and V-wave	Hmax/Mmax (%)		18.33 ± 11.98	(11.70, 24.97)
	Hth/Mth (%)		85.13 ± 8.63	(80.35, 89.91)
	H-index (cm^2^/ms^2^)		168.79 ± 23.50	(155.77, 181.80)
	V/MmaxA (%)		24.47 ± 18.61	(14.16, 34.77)
Muscle Strength	Hamstring (Nm/kg)	CON60°/s	1.77 ± 0.04	(1.68, 1.86)
		CON180°/s	1.52 ± 0.05	(1.40, 1.63)
		ECC60°/s	1.70 ± 0.07	(1.55, 1.85)
	Quadriceps (Nm/kg)	CON 60°/s	2.63 ± 0.11	(2.40, 2.86)
		CON180°/s	2.23 ± 0.13	(1.95, 2.52)
		ECC60°/s	2.80 ± 0.16	(2.47, 3.14)
	Gastrocnemius (Nm/kg)	CON 60°/s	1.68 ± 0.08	(1.51, 1.85)
		CON180°/s	1.26 ± 0.04	(1.17, 1.34)
		ECC60°/s	2.62 ± 0.12	(2.37, 2.87)
	H/Q (%)	CON 60°/s	68.60 ± 11.84	(62.04, 75.16)
		CON180°/s	70.46 ± 14.63	(62.36, 78.56)
		ECC60°/s	60.73 ± 11.71	(54.25, 67.22)
Stiffness	Achilles tendon stiffness (Nm/cm)		55.05 ± 13.28	(47.70, 62.41)
	Vertical stiffness (N/m/kg)		36.84 ± 10.03	(31.28, 42.40)
Lower limb explosive strength	30 m sprint time(s)		4.15 ± 0.15	(4.07, 4.24)
	RFD (N/s/kg)		50.00 ± 23.04	(37.85, 56.71)

Note: CON, concentric; ECC, eccentric.

Table 2 shows the correlation between the H-reflex and V-wave, skeletal muscle function, and lower limb explosive strength. 30 m sprint time significantly positively correlated with Achilles tendon stiffness (*r* = 0.50, *p* = 0.05, 95%CI: 0.01–0.81). H-index (*r* = −0.54, *p* = 0.04, 95%CI: 0.82 to −0.03), V/MmaxA (*r* = −0.59, *p* = 0.02, 95%CI: 0.85 to −0.11), and HECC60 (*r* = −0.53, *p* = 0.04, 95%CI: 0.82 to −0.03) showed significant negative correlations with 30 m sprint time, indicating that higher values of these indicators are associated with shorter 30 m sprint time. There was no significant correlation between the 30 m sprint time and concentric isokinetic knee flexion and extension (*p* > 0.05). RFD had a significant positive correlation with Hmax/Mmax (*r* = 0.57, *p* = 0.03, 95%CI:0.08–0.84) and QECC60 (*r* = 0.53, *p* = 0.04, 95%CI: 0.02–0.82).

**TABLE 2:** Correlation between the H-reflex and V-wave, skeletal muscle function, and lower limb explosiveness.

	30 m sprint time(s)		RFD (N/s/kg)	
	*r*	95%CI	*r*	95%CI
Hmax/Mmax	−0.30	(−0.70, 0.25)	0.57*	(0.08, 0.84)
Hth/Mth	0.40	(−0.14, 0.76)	0.18	(−0.36, 0.64)
H-index	−0.54*	(−0.82, −0.03)	−0.13	(−0.60, 0.41)
V/MmaxA	−0.59*	(−0.85, −0.11)	0.28	(−0.27, 0.69)
HCON60	−0.28	(−0.69.0.27)	−0.19	(−0.64, 0.35)
QCON60	−0.17	(-0.63, 0.38)	0.27	(-0.28, 0.69)
H/QCON60	−0.05	(−0.55.0.48)	−0.32	(−0.71, 0.23)
HCON180	−0.19	(−0.64.0.35)	−0.21	(-0.65, 0.34)
QCON180	−0.25	(−0.68.0.30)	0.12	(−0.42, 0.59)
H/QCON180	0.10	(−0.44.0.58)	−0.27	(−0.69, 0.27)
HECC60	−0.53*	(−0.82, −0.03)	0.16	(−0.39, 0.62)
QECC60	−0.03	(−0.62, 0.39)	0.53*	(0.02, 0.82)
H/QECC60	−0.31	(−0.71, 0.24)	−0.28	(−0.69, 0.27)
GMCON60	0.48	(−0.04, 0.80)	−0.37	(−0.74, 0.18)
GMCON180	0.21	(−0.34, 0.65)	−0.48	(−0.80, 0.04)
GMECC60	0.29	(−0.27, 0.70)	−0.29	(−0.70.0.26)
Achilles Tendon stiffness	0.50*	(0.01, 0.81)	−0.32	(−0.72, 0.23)
Vertical stiffness	0.03	(−0.49, 0.54)	0.83**	(0.55, 0.94)

Note: *p ≤ 0.05; **p ≤ 0.01; H, hamstring; Q, quadriceps; GM, gastrocnemius; CON, concentric; ECC, eccentric; 60, 60°/s; 180, 180°/s.

Table 3 shows the results of the stepwise multiple regression analysis of predictors determining 30 m sprint time. V/MmaxA and HECC60 were identified as predictors of 30 m sprint time, and the *R* ^2^ explained 57.5% of the variance. The multivariate formula was: 30 m sprint time = 4.713–0.448*V/MmaxA −0.264*HECC60. Vertical stiffness and QECC60 explained 82.7% of the variation in RFD.

**TABLE 3:** Stepwise multiple regression analysis of predictors that influenced 30 m sprint time and RFD.

Dependent variable	Predictors	Unstandardized coefficients		Standardized coefficients	t	Sig	95% CIfor B
		B	Std. error	Beta			
30 m sprint time	(Constant)	4.713	0.181	-	26.034	<0.001	
	V/MmaxA	−0.448	0.156	−0.542	−2.862	0.014	−0.788,-0.107
	HECC60	−0.264	0.105	−0.476	−2.515	0.027	−0.493,-0.035
	*R* ^2^	0.575					
	Adjusted *R* ^2^	0.504					
	F	F (2.12) = 8.112, *p* = 0.006					
	D-W value	2.648					
RFD	(Constant)	−29.819	11.283	-	−2.643	0.021	
	Vertical Stiffness	1.282	0.208	0.756	6.164	<0.001	0.829.1.735
	QECC60	10.647	3.454	0.378	3.083	0.009	3.122.18.172
	*R* ^2^	0.827					
	Adjusted *R* ^2^	0.798					
	F	F (2.12) = 28.585, *p* < 0.001					
	D-W value	1.500					

## 4 Discussion

The primary findings of this study indicated that V/MmaxA and HECC were predictive factors of 30 m sprint time and vertical stiffness and QECC was the predictive factor of RFD. Consistent with this study’s hypothesis, neural factors have a greater impact on lower limb explosive strength, V/MmaxA, Hmax/Mmax, and NCV were significantly correlated with 30 m sprint time and RFD.

Sprinters need to react rapidly and accelerate to a high horizontal speed from a stationary position after receiving an external order, which requires a high-level neuromuscular function ability (Bezodis et al., 2019). 30 m test is commonly used to assess acceleration and lower limb explosive strength, and this study found that neural and muscular factors were both associated with 30 m sprint time (Healy et al., 2022; Samozino et al., 2022). Recent studies have revealed that lower limb explosive strength is determined by the ability of the neural drive to the muscle, especially driven by the cortex (Del et al., 2019; Grospretre et al., 2018). Rapid contractions of muscles depend on motor units that are highly synchronized to activate and a high initial discharge rate at the onset of activation (Maffiuletti et al., 2016; Del, 2023). V-wave was considered an indicator evaluating the neural drive in descending corticospinal pathways, reflecting the change of motoneuron excitability and neural adaptation to training (Aagaard et al., 2002; Nevanpera et al., 2023; Tomazin et al., 2022). This study found that the V-wave was a key determinant of 30 m, demonstrating that a large magnitude of descending central drive from supraspinal centers may enhance sprint performance. Similar results have also been observed in female ice hockey players: V-wave amplitude increased significantly, accompanied by an increase in plantarflexion MVC force and RFD after around 2 weeks of HIIT training (Kinnunen et al., 2019b). Meanwhile, the results showed no significant correlation between the H-reflex and 30 m in sprinters. Compared to endurance athletes, a lower H-reflex was observed in sprinters (Maffiuletti et al., 2001; Ozmerdivenli et al., 2002; Tøien et al., 2023). After short-term endurance and strength training, V/MmaxA significantly increased in the strength group, with no changes in the endurance group, and H/M displayed reverse trends (Vila-Chã et al., 2012). This indicates that changes following training in the strength group depend on the increased descending neural drive instead of spinal α-motoneuron excitability, leading to increased MVC. Strength-trained athletes demonstrated a larger V-wave than healthy adults (Tøien et al., 2023). Therefore, V waves increased after training may be beneficial for improving lower limb explosive strength.

This study also showed a significant moderate negative correlation between H-index and 30 m sprint time. NCV reflects the velocity transmitted along a motoneuron of the impulse, while rapid NCV may accelerate muscle contraction, leading to a higher speed in 30 m (Domkundwar et al., 2017; Vonbank et al., 2023). Research investigating NCV in sprint performance is limited. A study of the posterior tibial NCV of athletes indicated that male sprinters have higher NCV than marathoners and lower NCV than distance runners and weight lifters (Kamen et al., 1984). In addition, one study found that the reaction time strongly correlated with 100 m running time, finalists’ reaction time were shorter significantly than semifinalists in athletic world championships (Tonnessen et al., 2013). Higher NCV may shorten the reaction time, potentially helping sprinters shorten the 100 m running time (Crotty et al., 2022). The central neurotransmissions in auditory and sensorimotor systems were found to correlate with 100 m performance, and existing significant differences between elite and sub-elite adolescent sprinters (Hsieh et al., 2024). Thus, the excitability of motor neurons and nerve conduction in the cerebral cortex has a significant impact on improving sprint performance.

High levels of eccentric strength of the hamstring were found to strongly correlate with 30 m performance in this study, also as a predictor of 30 m sprint time, consistent with previous research. Hamstrings contract eccentrically as knee flexors to decelerate the momentum of the tibia and prevent knee hyperextension during the terminal swing phase, which may contribute to increasing step length and energy efficiency (Guex and Millet, 2013; Guruhan et al., 2021). Previous studies also found a large correlation between maximum velocity (Vmax) and isoinertial eccentric force (r = 0.56) in sprinters. The eccentric force can help sprinters adjust lower limb stiffness to exhibit high levels of reactive strength and maintain higher speeds (Douglas et al., 2020). Brady et al. (2020) reported that peak force, RFD and impulse of Isometric mid-thigh pull and Isometric squat significantly correlate with 0–5 m performance in sprinters. Only the peak force of IMTP correlated with 0–30 m (r = −0.595). Greater sprinters were observed to have larger muscle volumes of hamstrings, which strongly correlated with 40 m sprint time (*r* = −0.670, *p* < 0.01) (Nuell et al., 2021). Meanwhile, eccentric strength deficits in the hamstrings are common factors causing injury to sprinters, and enhancing eccentric strength training of the hamstrings may reduce the incidence of injury (Alt et al., 2021; Rudisill et al., 2023). A rehabilitation protocol consisting of eccentric exercises is more effective for elite sprinters with acute hamstring injuries, helping them return to training or competition quickly (Askling et al., 2014; Hickey et al., 2022).

The current results also showed a positive correlation between active tendon stiffness in the ankle joint and 30 m sprint time, inferring that relatively compliant tendons are beneficial for utilizing elastic energy. Tendon stiffness of plantar flexors was considered important enough to probably affect the storage and release of elastic energy, contributing significantly to propulsive and upward impulses (Aeles et al., 2018; Crotty et al., 2024). No significant differences in the active tendon stiffness of the plantar flexors were observed between sprinters and untrained individuals (Kubo et al., 2011; Kubo et al., 2017). Previous studies have found the Achilles tendon stiffness of sprinters during ballistic contractions is lower than during ramp contractions, which means the Achilles tendon stiffness of sprinters is more compliant when sprinting and jumping, similar to the results of this study (Kubo et al., 2020). The data predicted by a full-body musculoskeletal model showed that tendon compliance has minimal effort in sprint performance, a 10% increase in tendon compliance results in a 0.3% increase in maximum sprinting speed (Lin and Pandy, 2022). This study also indicates that tendon stiffness may not be a predictor of sprint performance. However, plantar flexor passive stiffness was negatively correlated with 100 m performance (r = −0.334, *p* = 0.018) in well-trained male sprinters (Takahashi et al., 2018). In addition, studies reported the correlation between other muscle-tendon parameters and sprint performance. The cross-sectional area and length of the Achilles tendon positively correlated with the running velocity and power of a 20 m sprint (Monte and Zamparo, 2019). The length of the Achilles tendon also has no correlation with 100 m performance (Tomita et al., 2021). The muscle volume of plantar flexors showed no differences between elite and sub-elite sprinters, but elite sprinters have higher muscle volume of hip extensors (Miller et al., 2022; Miller et al., 2021).

Sprinters need to apply vertical and horizontal forces to the ground rapidly to generate explosive contractions from a stationary position at the start; therefore, sprinters exhibit higher RFD and impulses (Slawinski et al., 2010). Previous studies found that vertical jump performance of top sprinters is correlated with top-speed phases, and the mean propulsive power in vertical jumps of elite sprinters is also correlated with sprint time (Loturco et al., 2018; Loturco et al., 2019). In this study, RFD correlated with Hmax/Mmax, eccentric strength of the quadriceps and vertical stiffness. Hmax/Mmax is an indicator of the α-motoneurons excitability from the Ia afferent and also reflects the stretch reflex, influencing the performance of the SSC (Leukel et al., 2008; Sun et al., 2022). CMJ requires the subject’s lower limb to start with an eccentric contraction, excited Ia afferent and increased spinal α-motoneurons excitability, leading to a large eccentric strength of the quadriceps. Eccentric contractions generate greater strength of the lower limb, making the subsequent concentric contraction faster and resulting in a higher RFD. Previous studies have shown that faster sprinters with higher vertical force achieve greater acceleration during the maximal speed phase (Nagahara et al., 2024; Nagahara et al., 2018). Sprinters reduce ground contact time by greater vertical stiffness, and their lower limbs are regularly subject to high-ground reaction forces (Maloney and Fletcher, 2021). A large difference between sprinters and untrained individuals in leg stiffness may be because the sprinter has a shorter ground contact time (Douglas et al., 2018). Greater vertical stiffness reflects improved neuromuscular control, helping sprinters move rapidly. Although the vertical stiffness significantly correlated with RFD in this study, RFD and vertical stiffness were both calculated by the peak force of the eccentric phase of the CMJ test. In the eccentric phase, shorter time leads to smaller ΔCOM, which may result in a spurious correlation and can be explored and validated further.

This study had several limitations. First, the sample size of this study is small because of limited access to elite male college sprinters, which limits statistical power and generalizability. Thus, these findings should be approached with caution. Further research is needed to increase the sample size and groups to explore the neuromuscular function of different-level sprinters. Second, the H reflex and M wave test in this study were in rest, the H-reflex and M-wave during muscle contraction could also be further explored, which better reflects neural modulation of actual movement. Third, this study only tested a 30 m sprint, without a 100 m test. Finally, the subjects were only male sprinters.

In summary, sprinters require strong neuromuscular control ability, and neural factors from the cortex are the primary determinants of lower limb explosiveness. H reflex and V-wave were sensitive to changes in neural function, which can be used to monitor changes in α-motoneurons excitability and assess the fatigue of the central and peripheral nervous system in a training period. In addition, NCV was found to correlate with a 30 m sprint performance, future research could explore the relationship between NCV and sports performance in higher-level sprinters.

## 5 Conclusion

This study found that V/MmaxA and HECC were predictive factors of 30 m sprint time, vertical stiffness and QECC were the predictive factors of RFD. Neural factors such as the α-motoneurons excitability of the spinal and supraspinal centers, have a greater influence on lower limb explosive strength in male college sprinters. Therefore, training related to the neural function of sprinters should be emphasized. In addition, H reflex and V wave can be used widely to assess and monitor the neural function of sprinters in future research. The impact of neural drive on muscles in different levels and sexes of sprinters, and the neuromuscular modulation during muscle contractions can be further explored.

## Data Availability

The raw data supporting the conclusions of this article will be made available by the authors, without undue reservation.
